# Supplementary material for: The causal role of circulating inflammatory markers in osteoporosis: a bidirectional Mendelian randomized study
Source: Front Immunol. 2024 Jul 18;15:1412298. doi: 10.3389/fimmu.2024.1412298 (PMC11291241; doi:10.3389/fimmu.2024.1412298)
Supplement: Supplementary file 1 [file DataSheet_1.zip › supplementary material/OSTEOPOROSIS-CIM/GCST90274833.tsv.gz_leave_one_out.pdf]

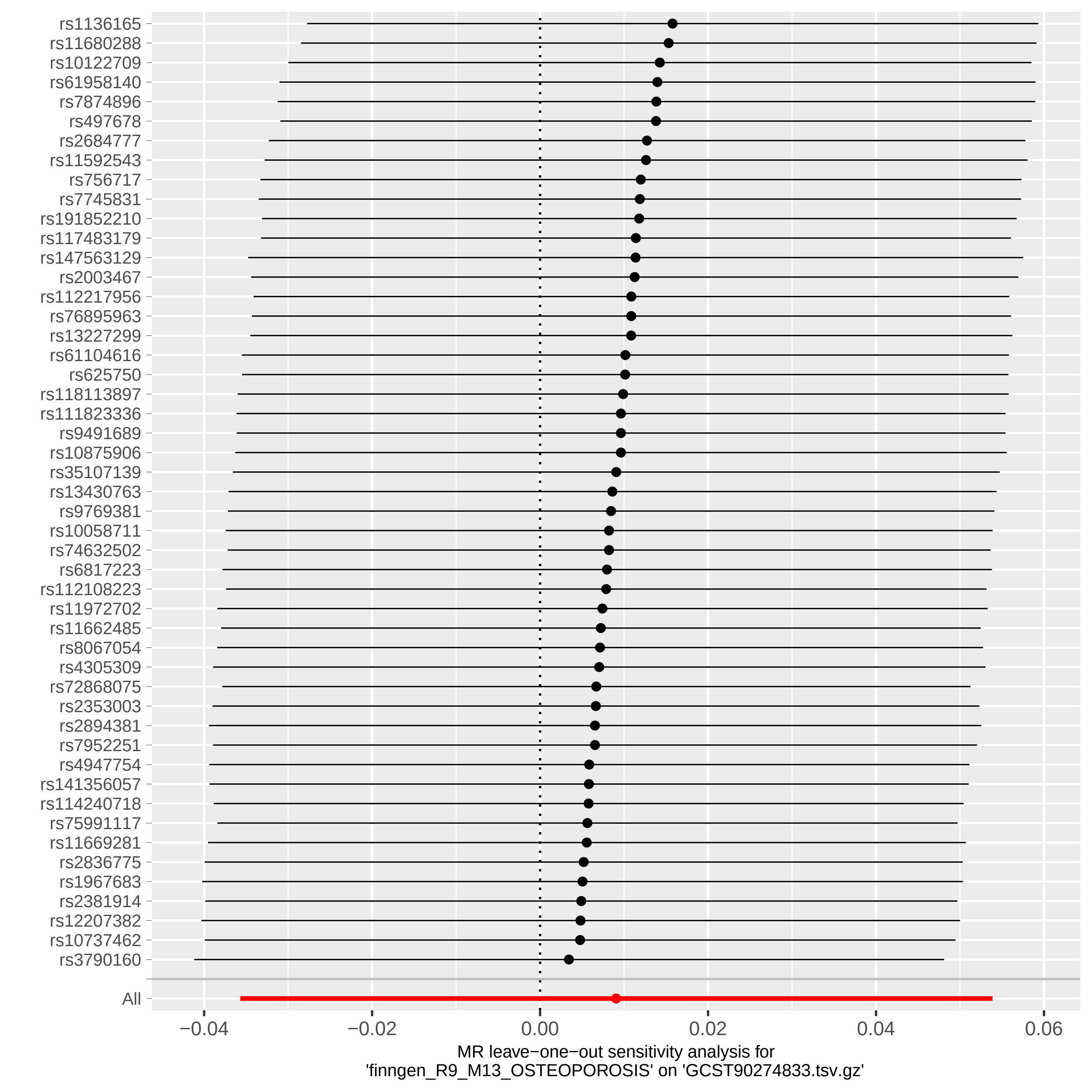
rs1136165
rs11680288
rs10122709
rs61958140
rs7874896
rs497678
rs2684777
rs11592543
rs756717
rs7745831
rs191852210
rs117483179
rs147563129
rs2003467
rs112217956
rs76895963
rs13227299
rs61104616
rs625750
rs118113897
rs111823336
rs9491689
rs10875906
rs35107139
rs13430763
rs9769381
rs10058711
rs74632502
rs6817223
rs112108223
rs11972702
rs11662485
rs8067054
rs4305309
rs72868075
rs2353003
rs2894381
rs7952251
rs4947754
rs141356057
rs114240718
rs75991117
rs11669281
rs2836775
rs1967683
rs2381914
rs12207382
rs10737462
rs3790160
All
−0.04
−0.02
0.00
0.02
0.04
0.06
MR leave−one−out sensitivity analysis for
'finngen_R9_M13_OSTEOPOROSIS' on 'GCST90274833.tsv.gz'
